# Cough: are children really different to adults?

**DOI:** 10.1186/1745-9974-1-7

**Published:** 2005-09-20

**Authors:** Anne B Chang

**Affiliations:** 1 Paediatric Respiratory and Sleep Physician, NHMRC Practitioner Fellow, Associate Professor in Paediatrics and Child Health, Dept of Respiratory Medicine, Royal Children's Hospital, Herston Rd, Brisbane, Queensland 4029, Australia

## Abstract

Worldwide paediatricians advocate that children should be managed differently from adults. In this article, similarities and differences between children and adults related to cough are presented. Physiologically, the cough pathway is closely linked to the control of breathing (the central respiratory pattern generator). As respiratory control and associated reflexes undergo a maturation process, it is expected that the cough would likewise undergo developmental stages as well. Clinically, the 'big three' causes of chronic cough in adults (asthma, post-nasal drip and gastroesophageal reflux) are far less common causes of chronic cough in children. This has been repeatedly shown by different groups in both clinical and epidemiological studies. Therapeutically, some medications used empirically for cough in adults have little role in paediatrics. For example, anti-histamines (in particular H_1 _antagonists) recommended as a front-line empirical treatment of chronic cough in adults have no effect in paediatric cough. Instead it is associated with adverse reactions and toxicity. Similarly, codeine and its derivatives used widely for cough in adults are not efficacious in children and are contraindicated in young children. Corticosteroids, the other front-line empirical therapy recommended for adults, are also minimally (if at all) efficacious for treating non-specific cough in children. In summary, current data support that management guidelines for paediatric cough should be different to those in adults as the aetiological factors and treatment in children significantly differ to those in adults.

## Introduction

To health care professionals who work with them, children are clearly different to adults but this seems less obvious to some. "Children swallow just like adults", remarked an academic speech pathologist when commenting on dysphagia and cough. "Children are the same as adults. It's just the behaviour that is different", remarked another specialist. Paediatricians world-wide passionately advocate that childhood illnesses should be managed differently to adults as extrapolation of adult based data to children can result in unfavourable consequences [1,2]. This article provides an update on paediatric issues on cough and highlights the differences between adults and children that are relevant to cough.

## Physiology

### Central and peripheral cough pathway

The central pathway for cough is a brainstem reflex linked to control of breathing (the central respiratory pattern generator) [3], which undergoes a maturation process such that the reference values for normal respiratory rate in children are different to those in adults [4] and reaches adult values in adolescence. In early life, cough is related to primitive reflexes (laryngeal chemoreflex), that undergo maturation resulting in significant differences in swallowing between young children and adults [5]. Plasticity (modulation) of the cough reflex has been shown [3,6], although it is unknown if the young have greater plasticity (propensity to modulate or change). Like other organs directly relevant to cough (eg the systemic and mucosal immune system) [7,8] or not directly related to cough (eg the renal system), one can speculate that the cough reflex has maturational differences as well. Indeed children differ from adults in some immunological response to lipopolysaccharides [9]. Also, children, especially their neurological system, are more sensitive than adults to certain environmental exposures [10]. For example, in children, the utility of CT scans has to be balanced with the reported increased lifetime cancer mortality risk, which is age and dose dependent. Although the risk is relatively negligible, children have 10 times increased risk compared to middle aged adults [10]. Lastly, the distinct differences in respiratory physiology and neuro-physiology between young children and adults include maturational differences in airway, respiratory muscle and chest wall structure, sleep characteristics, respiratory reflexes and respiratory control [11-13].

### Cortical control of cough and psychological determinants

Cough can be cortically modulated [14]. In adults, chronic cough is associated with anxiety as an independent factor [15]; such data are unavailable in children. Adults seeking medical attention are primarily self-driven but in children, parental and professional expectations influence consulting rates and prescription of medications [16-18]. Reporting of childhood respiratory symptoms is biased and parental perception of childhood cough plays an important role [19,20]. In asthma, parental psychosocial factors (in particular anxiety) were strongest predictors for emergency attendances for children whereas in adults, asthma severity factors were the risk factors [21]. In cough, use of cough medications and presentation to doctors were less likely in children with higher educated mothers [22]. Hutton and colleagues' described "parents who wanted medicine at the initial visit reported more improvement at follow-up, regardless of whether the child received drug, placebo, or no treatment" [23]. Rietveld and colleagues showed that children were more likely to cough under certain psychological settings [24,25].

## Clinical evaluation of cough

### What is 'normal' or expected?

'Normal' children occasionally cough as described by two studies that objectively measured cough frequency [26,27]. Normal children without a preceding upper respiratory infection in the last 4 weeks have up to 34 cough epochs per 24 hours [26]. In another study, 0–141 cough epochs/24 hours (median 10) were recorded in 'controls' (these children were considered well by parents and attending school and were age, gender and season matched [27]). Medicalisation of an otherwise common symptom can foster exaggerated anxiety about perceived disease and lead to unnecessary medical products and service [28]. Cough in this situation is termed 'expected cough'. Such data are unavailable in adults.

However, concerns of parents presenting to general practitioners for their children's cough can be extreme (fear of child dying, chest damage) [29,30]. Other parental concerns were disturbed sleep and relief of discomfort [29]. However the burden of illness on children and their family has not been well described. In contrast adult data have shown that chronic cough causes a significant burden of illness (physical and psychosocial) that is often not appreciated by physicians [20] as reflected in adult cough-QOL scores [31,32].

### What is acute and what is chronic?

The utility of definitions depends on the intention of use. In adults, chronic cough is defined as cough lasting >8 weeks [33]. In children the definition of chronic cough varies from 3-weeks duration [34] to 12-weeks [35,36]. There are no studies that have clearly defined when cough should be defined chronic or persistent. As studies have shown that cough related to ARIs resolves within 1 to 3 weeks in most children [17,37] it would be logical to define chronic cough as daily cough lasting >4 weeks.

### Classification of paediatric cough

Paediatric cough can be classified in several ways, based on aetiology [38], timeframe [35] and characteristic (moist vs dry). For practical reasons, guidelines based on cough duration, combined with cough quality have been developed [35]. An evidence based guideline specific for paediatrics will be published as part of the American College of Chest Physicians' Guidelines on the Management of Cough in Adults and Children [39]. The previous guidelines which stated that "the approach to managing cough in children is similar to the approach in adults" [34] was arguably inaccurate.

**Figure 1:**
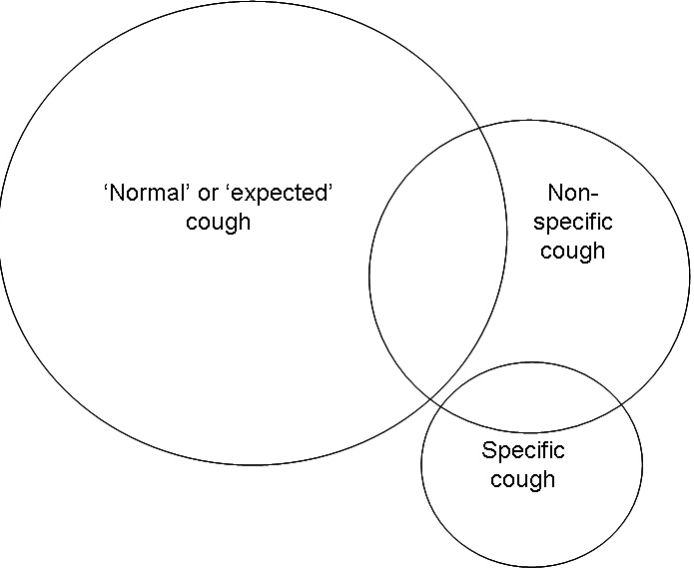
Classification of types of cough in children (reproduced from [110]).

Unlike cough in adults, paediatric cough has also been classified into specific and non-specific cough (with an overlap) for practical reasons (figure 1). Indeed, the most common paradigm encountered in clinical paediatrics when cough is a presenting feature is the differentiation between specific and non-specific cough. Specific cough refers to cough in the presence of pointers (table 1) that suggest the presence of an underlying aetiology. A thorough history and examination to elucidate these points are necessary when assessing children with cough and in the majority of situations, specific cough aetiologies can be defined. While some of these symptoms and signs are common in adults (such as haemoptysis), others are not (such as failure to thrive). Unlike in adults, where cough characteristics has been shown to be of little diagnostic value [40], paediatricians often recognise certain cough qualities such as staccato cough (table 2). A chronic moist cough is always abnormal and represents excessive airway secretions [41]. However in a small group of children natural resolution may occur [42] and a specific paediatric diagnostic category may not be found [43]. A chronic dry cough however may represent a dry phase of an otherwise usually moist cough or airway secretions too little to influence the cough quality [41]. Chronic dry cough in the absence of specific pointers (table 1) in the history and examination is termed 'non-specific cough' or 'isolated cough', ie cough is the sole symptom. In non-specific cough, the aetiology is ill defined and we suspect that the majority are related to post viral cough and/or increased cough receptor sensitivity [44,45]. However in the majority of children, it is most likely related to a non serious aetiology [38] or may spontaneously resolve as evidenced in the placebo arms of RCTs [46-48] and cohort studies [49-51]. Thus if one assumes that the natural resolution of non-specific cough occurs in 50% of children, 85 children per study arm is required in a randomised controlled trial to detect a 50% difference between active and placebo groups, for a study powered at 90% at the 5% significance level.

**Table 1:** Pointers to underlying aetiology i.e. presence of specific cough [39,110]

auscultatory findings
cough characteristics eg cough with choking, cough quality (table 2), cough starting from birth
cardiac abnormalities (including murmurs)
chest pain
chest wall deformity
chronic dyspnoea
daily moist or productive cough
digital clubbing
exertional dyspnoea
failure to thrive
feeding difficulties
haemoptysis
immune deficiency
neurodevelopmental abnormality
recurrent pneumonia

**Table 2:** Classical recognisable cough [39,110]

Barking or brassy cough	Croup [252] tracheomalacia [132,134] habit cough [157,253]
Honking	Psychogenic [254]
Paroxysomal (with/without whoop)	Pertussis and parapertussis [123,255]
Staccato	Chlamydia in infants [256]
Cough productive of casts	Plastic bronchitis [257]

### Symptoms

#### Nocturnal cough

In both adults and children, a major problem in utilising the symptom of nocturnal cough is the unreliability and inconsistency of its reporting when compared to objective measurements [52-54]. In children, however, two groups have reported that parents were able to detect change [46,54], albeit only moderately well. The ability to detect cough change was better in children with a history of troublesome recurrent cough (r = 0.52) than in children without (r = 0.38) [54]. Relationship between change in cough frequency and change in subjective scores has not been examined in adults.

Nocturnal cough is often used as a hallmark of asthma as children with asthma often report troublesome nocturnal cough [55]. However in a community based study, only a third of children with isolated nocturnal cough had an asthma-like illness [56]. To date there are no studies that have objectively documented that nocturnal cough is worse than daytime cough in children with unstable asthma. One study showed that cough frequency was higher during the day than at night in a group of children with stable asthma who were on ICS yet had elevated levels of eNO but not sputum eosinophils [57] (arguably the best marker for eosinophilic inflammation in stable asthma [58]) in schoolchildren. Whether the increased eNO is a marker of asthma instability or related to other causes of elevated nitric oxide (such as environmental pollutants) [59,60] is unknown. Nocturnal cough has been reviewed elsewhere [61].

#### Cough quality

Unlike adults, cough quality is associated with specific aetiology in children (table 2). Except for brassy cough and wet cough, the sensitivity and specificity of cough quality have not been defined [62]. Thus perceived cough quality by parents and clinicians may have limitations. Pertussis-like cough in children may indeed be caused by adenovirus, parainfluenza viruses, respiratory syncytial virus and Mycoplasma [63]. Children with a dry cough are more likely to naturally resolve than those with wet cough [64]. Young children rarely expectorate even when airway secretions are excessive. Hence wet/moist cough is often used interchangeably with productive cough [65,66] a term used in adults. We have recently shown the clinical validity of dry and wet/moist cough in children by scoring secretions seen during bronchoscopy [41]. In contrast, quality of cough has been shown to be of little use in adults [40,67].

### Investigations

Children with specific cough usually require a variety of investigations which include chest CT, bronchoscopy, barium meal, video fluoroscopy, nuclear scans, sweat test, etc. The role of these tests for evaluation of lung disease is beyond the scope of this article as it would encompass the entire spectrum of paediatric respiratory illness. The more common problem of non-specific cough is further briefly discussed. In general investigations are rarely needed in non-specific cough.

#### Airway cellular assessment

Examination of cellular profile of induced sputum, a standard in some adult cough clinics, can only be performed in older children (children >6 years). The majority of children with chronic cough seen by paediatricians are in the toddler age group (1–5 years) where bronchoscopy is necessary to obtain airway cells. In contrast to adult studies, all 4 paediatric studies [51,68-70] that have examined airway cellularity in children with chronic cough have rarely found an asthma-like profile. Other than assessment of airway specimens for microbiological purposes, the use of airway cellular and inflammatory profile in children with chronic cough is currently entirely limited to supportive diagnosis and research rather than definitive diagnosis. This is in contrast to that in adults with chronic cough where some have suggested use of airway inflammatory profiles to direct therapy [71,72]. One study in children with 'cough variant asthma' (mean age 11 years) showed that those with a higher percentage (>2.5%) of eosinophils in their sputum were more likely to develop classical asthma on follow-up [73]. There was however no appropriate control group and sputum ECP was unpredictive of asthma [73].

#### Cough sensitivity measures

In the physiology of cough, gender differences in CRS well recognised in adults [74], are absent in children [44]. In children, CRS is instead influenced by airway calibre and age [44]. An adult type approach to CRS measurement that is reliant on a child inhaling and maintaining an open glottis during actuation of a dosimeter or during nebulisation is unreliable. Furthermore it has been shown in both adults [75,76] and children [77] that inspiratory flow (which influences lung deposition) influences CRS. Thus in children, regulation of a constant inspiratory flow is necessary for valid results [77]. Increased CRS has been found in children with recurrent cough [44], cough dominant asthma [78] and influenza infection [79]. However testing for CRS is non-diagnostic and its use is still limited to research purposes. In clinical circles, the concept of a temporal increase in CRS has been useful to explain 'expected cough'.

#### Use of chest and sinus CT scans

The utility of a CT scan in children has to be balanced with the reported increased lifetime cancer mortality risk [10]. The yield of ultrafast CT scans in children with chronic productive cough is 43%, where bronchiectasis was documented [80]. The yield of CT scan in evaluation of a dry cough without the presence of features in table 1 is unknown and arguably should not be performed. Lung cancers are extremely rare in children. In children, there is poor concordance in diagnostic modalities for diagnosing paranasal disease [81]. Also, a single study of paranasal sinus CT findings in children with chronic cough (>4 weeks) described that an abnormality was found in 66% [82]. However this finding has to be interpreted in the context of high rates (50%) of incidental sinus abnormality in asymptomatic children undergoing head CTs [83]. Abnormal sinus radiographs may be found in 18–82% of asymptomatic children [84]. Thus, it is arguably difficult to be confident of an objective diagnosis of nasal space disease as the cause of cough.

#### Flexible bronchoscopy

Indications for bronchoscopy in children with chronic cough include suspicion of airway abnormality, persistent changes on CXR, suspicion of an inhaled foreign body, evaluation of aspiration lung disease and for microbiological and lavage purposes. In these situations, cough is usually specific rather than non-specific. Bronchoscopically defined airway abnormality was present in 46.3% of children with chronic cough in a tertiary centre-based study, whereas in Callahan's [85] series, bronchoscopy assisted in diagnosis in 5.3% of children [86]. In a European series, chronic cough was the indication in 11.6% of the 1233 paediatric bronchoscopies performed [87].

#### Spirometry

Spirometry is valuable in the diagnosis of reversible airway obstruction in children with chronic cough. In the early studies on asthma presenting as chronic cough, abnormal baseline lung function was documented [88,89]. However spirometry is relatively insensitive [90,91] and a normal spirometry does not exclude underlying respiratory abnormality. In one study of 49 children with chronic cough, spirometry was normal in all who were able to perform the test [86].

#### Tests for airway hyper-responsiveness

In adults, tests for AHR are relatively easy to perform and direct AHR (methacholine, histamine) is used to exclude asthma [33]. In children (outside a research setting) testing for AHR is reliably performed only in older children (>6 years) and positive AHR especially to direct AHR challenges as an indicator of asthma has questionable validity [92,93]. Airway cellularity (sputum) in asymptomatic children with AHR was similar to children without AHR but significantly different to children with asthma [94]. In children, unlike in adults, the demonstration of AHR in a child with non-specific cough is unlikely to be helpful in predicting the later development of asthma [95] or the response to asthma medications [47]. The only RCT that examined the utility of AHR and response to inhaled salbutamol and ICS [47] found that the presence of AHR could not predict the efficacy of these therapies for cough [47]. Another study showed that AHR to hypertonic saline is significantly associated with wheeze and dyspnoea but not associated with dry cough or nocturnal cough once confounders were accounted for [96]. The older studies that equated presence of AHR in children with cough as representative of asthma were not placebo-controlled studies, confounders were not adjusted for, or used unconventional definitions of AHR [97-100]. A recent study using 6 min free running test described that exercise induced symptoms were poor predictors of bronchoconstriction [101]. However interpretation of the study is limited [102].

#### Other investigatory techniques

The single study on bronchial biopsies in 7 children with chronic cough described the association between early ARI and epithelial inflammation [103]. Bronchial biopsies are easily performed in adults, but are rarely performed in children except in selected centres where the procedure has been shown to be safe [104]. Airways resistance by the interrupter technique (Rint) has been used to asses values in children with cough [105] but Rint is not established in clinical practice and has problems with validity of measurements when undertaken by different investigators [106]. To date, there are no paediatric studies that have evaluated the role of NO or breath condensate in guiding management of chronic cough. Increased NO has been found in asthmatics with cough [57] but is also found in other conditions associated with cough such as environmental pollutants [60].

### Outcome measures for cough-related studies

Cough severity indices, broadly divided into subjective and objective outcomes, measure different aspects of cough. In children, measures of CRS have a weak relationship with cough frequency. Subjective cough scores have a stronger and consistent relationship with cough frequency [107]. The choice of indices depends on the reason for performing the measurement [107].

Answers to questions on isolated cough are largely poorly reproducible [108] and nocturnal cough in children is unreliably reported [52,53]. The kappa value relating the chance-corrected agreement to questions on isolated cough is poor (0.02–0.57) [19,108,109] in contrast to isolated wheeze (0.7–1.0) [108]. Biased reporting of cough has been shown; parents who smoke under-report cough in their children [19]. Diary cards for cough have been validated against an objective method and children aged >6 years are better than their parents at quantifying their cough severity [54]. Cough-specific QOL questionnaires exist for adults but not for children. There is a clear need for a paediatric cough specific QOL scores, as adult QOL scores cannot be applied to children. Cough specific objective tests include ambulatory and non-ambulatory objective cough meters, CRS and cough peak flows (reviewed elsewhere) [110]. Adult type instruments require modification for use in children [111].

### Aetiological factors

Although some diseases are common to both adults and children, the pattern of many respiratory illnesses in children is clearly different to adults; eg viruses associated with the common cold in adults can cause serious respiratory illnesses such as bronchiolitis and croup in previously well young children [112]. Both of these respiratory syndromes are non existent in adults. Conversely, common causes of cough and respiratory diseases in adults such as chronic bronchitis [113] and chronic obstructive pulmonary disease are not recognised diagnostic entities in paediatric respiratory literature and main textbooks [114,115]. The following highlights some of the differences between children and adults.

#### Cohort studies

Some hospital based clinical studies of children presenting with chronic cough have found asthma as the most common cause [116,117] but others have not [43,86]. In a prospective review of 81 children with chronic cough, none had asthma on final diagnosis [43]. In a retrospective review of 49 children with chronic cough, none of the children had asthma as the sole final diagnosis [86]. There is little doubt that the aetiology of cough would depend on the setting, selection criteria of children studied [69,86] follow-up rate [118] and depth of clinical history, examination and investigations performed. When airway profiles have been examined in children with isolated chronic cough, the studies have shown very few children with airway inflammation consistent with asthma [68-70]. Marguet and colleagues concluded that "chronic cough is not associated with the cell profiles suggestive of asthma and in isolation should not be treated with prophylactic anti-asthma drugs" [70].

#### Acute respiratory infections and post infections

Most coughs in early childhood are caused by viral ARIs [17,119]. In children with an ARI, 26% were still unwell 7-days after the initial consultation and 6% by day 14 [120]. Cough was however not specifically reported [120]. A systematic review on the natural history of acute cough in children aged 0–4 years in primary care reported that the majority of children improve with time but 5–10% progress to develop bronchitis and/or pneumonia [17]. Post-viral cough is a term that refers to the presence of cough after the acute viral respiratory infection. In Monto's review [121] the mean annual incidence of total respiratory illness per person year ranges from 5.0–7.95 in children aged less than 4 years to 2.4–5.02 in children aged 10–14 years [121]. A recent Australian study recorded respiratory infection/episode rates of 2.2–5.3 per person per year for children aged ≤10 years (mean duration of 5.5–6.8 days) [122]. That for adults (>20-years) was 1.7 [122].

Infections such as pertussis and mycoplasma can cause persistent cough not associated with other symptoms [123]. Pertussis should be suspected especially if the child has had a known contact with someone with pertussis even if the child is fully immunised as partial vaccine failure is an emergent problem [124]. A hospital study examined PCR and serology for pertussis in a prospective cohort of 40 children with chronic (>3 weeks) cough and found that only 5% of these children had laboratory evidence of pertussis [42]. No other published data on chronic cough have examined pertussis and mycoplasma infections with other cough etiologies. In a prospective childhood vaccine study, presence of Chlamydia pneumoniae, mycoplasma, parapertussis and pertussis were sought in children (aged 3–34 months) if a child or household member coughed for >7 days. In total, 115 aetiological agents were identified in 64% of episodes with cough [123]. The most common single agent was pertussis in 56% (median cough of 51 days), followed by Mycoplasma in 26% (cough for 23 days), Chlamydia in 17% (26 days), and parapertussis 2% [123]. Other microbial studies were not done. A factor that needs to be considered when analysing such results is determining whether the infectious agent isolated is the cause of the cough, as the percentage of asymptomatic infection can be very high (54%) [125]. In children who received the acellular pertussis vaccination, pertussis infection is clinically difficult to distinguish from diseases associated with coughing caused by other viral or bacterial infections [126].

#### Inhalation of foreign body

Cough is the most common symptom in some series of acute foreign material inhalation but not in others [127]. A history of a choking episode is absent in about half [128]. Presentations are usually acute [129] but chronic cough can also be the presentation of previously missed foreign body inhalation [130]. Unlike adults, a history of acute aspiration in young children has to be obtained from an adult who may not be present at the time of aspiration. Missed foreign bodies in the airways can lead to permanent lung damage [131].

#### Airway lesions and cough

Chronic cough is well described in children with airway lesions [132-134] and at lesser frequency in adults [135]. An adult study reported that none of 24 patients with tracheomalacia had chronic cough as a presenting symptom [135]. Gormley and colleagues described that 75% of children with tracheomalacia secondary to congenital vascular anomalies had persistent cough at presentation [134]. Other symptoms include stridor, chronic dyspnoea, recurrent respiratory infections and dysphagia [134]. How common are airway lesions in asymptomatic children is unknown and how the symptom of cough relates to airway lesions can only be postulated.

#### Environmental pulmonary toxic agents

In-utero tobacco smoke exposure alters respiratory control and responses [136,137], pulmonary development and physiology [138,139]. Its influence on the developing central and peripheral cough receptors, pathways and plasticity of the cough pathway [6,140] is unknown. ETS increases susceptibility to respiratory infections [141,142] causes adverse respiratory health outcomes [143] and increases coughing illnesses [144,145]. Increased ETS has also been described in cohorts of children with chronic cough compared to children without cough [69,69,143,144,146,147]. Indoor biomass combustion increases coughing illness associated with acute respiratory infections with an exposure-response effect [148]. Exposure to other ambient pollutants (particulate matter [149,150] nitrogen dioxide, gas cooking [151] etc) is also associated with increased cough in children in cross sectional [149,150] and longitudinal studies [152] especially in the presence of other respiratory illnesses such as asthma [149]. Some studies however have not shown this effect [153,154] which is likely partially related to problems with question-based epidemiological studies on isolated and nocturnal cough [14,19].

#### Functional respiratory disorder

Habitual cough or cough as a 'vocal tic' maybe transient or chronic and are far more commonly reported in the paediatric literature than in the adult literature [41]. In one series, psychogenic cough accounted for 10% of children with chronic cough [116]. A Swedish community study described the prevalence of chronic vocal tics was 0.3% in girls and 0.7% in boys [155]. The cough in psychogenic cough is typically thought to be absent at night. However objective cough recording in a child with psychogenic cough showed that cough during sleep does occur [156]. The typical psychogenic cough (honking cough) recognisable in children [67,157] is rare in adults [67]. In one study, 52% of those who had their cough recorded had barking (brassy, croupy) or honking cough [158]. However, brassy or croupy cough is also found in other childhood conditions associated with cough such as tracheomalacia [41].

#### The big three of chronic cough in adults

In adults, asthma, GORD, post-nasal drip (the big three) are said to cause upto 72–90% of chronic cough [159,160]. In contrast, there is no good data that suggest that these are common causes of chronic cough in children.

##### Asthma, reactive airway disease and cough in children

There is little doubt that children with asthma may present with cough. However, the majority of children with cough do not have asthma [14,69,70,161,162]. The use of isolated cough as a marker of asthma is indeed controversial with more recent evidence showing that in most children, isolated cough does not represent asthma [35,162]. Cough associated with asthma without a co-existent respiratory infection is usually dry [163]. Some medium term cohort studies on children with cough have suggested that the majority of these children eventually developed asthma [73,164] but other studies have not [49,50,165,166]. The Tuscon group showed that recurrent cough presenting early in life resolved in the majority [166]. Furthermore, these children with recurrent cough and without wheeze, had neither AHR nor atopy, and significantly differed from those with classical asthma, with or without cough [166]. Several other studies also support McKenzie's annotation [161] which highlighted the problem of over-diagnosis of asthma based on the symptom of cough alone [118]. In a prospective community study with a mean follow-up period of 3 years, 56% of children with recurrent cough aged 4–7 years later became asymptomatic; 37% reported continuing cough and 7.2% developed wheeze [49]. The proportion of children in the group who subsequently developed wheeze was similar to the asymptomatic group, who later developed wheeze on follow-up (10%) [49]. Faniran and colleagues concluded in their community based study of 1178 children that "cough variant asthma is probably a misnomer for most children in the community who have persistent cough" [118]. Thus in community settings, epidemiological studies have shown that isolated persistent cough is rarely asthma [118,161,165,167]. These data have been previously reviewed [14].

##### Upper airways disorders and cough in children

In adults, post-nasal drip has been reported as a common cause of cough [40]. In children, although nasal discharge and cough have been reported as the two most prominent symptoms in children with chronic sinusitis (30–120 days) [168] supportive evidence of cause and effect in children is less convincing [169]. A prospective study has shown that although sinusitis is a common condition in childhood, it is not associated with asthma or cough when the confounding factor of allergic rhinitis was removed [170]. The relationship between nasal secretions and cough is more likely linked by common aetiology (infection and/or inflammation causing both) or due to clearing of secretions reaching the larynx. Using a continuous infusion of 2.5 mls/min of distilled water into the pharynx of well adults, Nishino and colleagues demonstrated that laryngeal irritation and cough only occurred in the presence of hypercapnia (45–55 mmHg) [171] suggesting that pharyngeal secretions alone do not cause cough. Physiologically this is to be expected as the pharynx is not innervated by the vagus nerve, a necessary component of the cough reflex [172]. One study described increased extrathoracic AHR without bronchial AHR to methacholine in a group of children presenting with chronic cough [173] and other studies have linked extrathoracic AHR to sinusitis and rhinitis [174,175]. However, the repeatability and validity of extrathoracic AHR in children are ill-defined. Therapeutic approaches for allergic rhinitis have been well summarised [176].

##### GOR and cough in children

In adults, GORD is reported to cause up to 41% of chronic cough [177]. In non-controlled trials the improvement rate of cough by non-surgical intervention e.g. with PPI alone [178] or PPI with motility agents [179] for GORD associated cough, cough improvement rates of 86–100% have been reported [178,179]. However a systematic review found much less convincing results [180]. In children the data relating isolated cough to GORD is even far less convincing. The section on upper airway symptoms of a clinical practice guideline on the evaluation and management of children with GOR included a discussion on cough and GOR, concluded "...there is insufficient evidence and experience in children for a uniform approach to diagnosis and treatment" [181]. Cough unequivocably (RCT setting) related to acid GOR in adults has been reported to subside in 1–3 weeks [182] but such evidence is unavailable in children [180] and difficult to obtain. While GOR may be the reason for persistent cough [183,184] cough can also cause GOR [185,186]. Proof of cause and effect in children is rare [187] and it is difficult to delineate cause and effect [188]. There are limited studies which have prospectively examined causes of chronic cough in children. Those available suggest GOR is infrequently the sole cause of isolated cough in children. One prospective study of the causes of chronic cough in children found only one child with GOR out of a series of 38 [116]. A retrospective study found co-existent GOR in 4 of 49 children with chronic cough [86]. In contrast to data in adults where GOR is a frequent cause of chronic cough [159,189] there is indeed no current convincing evidence that GOR is a common cause of non-specific cough in children. Although case series have shown the link between supra-oesophageal reflux and GOR in children, there is a lack of convincing data, as Rudolph summarised "No studies have definitively demonstrated symptom improvement with medical or surgical therapy for the latter symptom presentations" [190].

#### Other aetiologies

##### Eosinophilic bronchitis and allergy

Eosinophilic bronchitis, a well described cause of chronic cough in adults [191] is not well recognised in children. 'Allergic or atopic cough' is a poorly defined condition even in adults [192]. The association between atopy and respiratory symptoms has been the subject of many epidemiological studies [193,194]. Some have described greater respiratory symptom chronicity [195] but others have not [193,194]. Inconsistent findings regarding cough and atopy are also present in the literature; reports of increased atopy (or diseases associated with atopy) in children with cough have been found in some cohort and cross sectional studies [165,196] but not in others [46,47,56,166]. Cough as a functional symptom can also be mistaken for an allergic disorder in children [197].

##### Medications and treatment side-effects

Chronic cough has been reported as a side effect of ACE inhibitors (2–16.7%) [198-200], inhaled ICS [201] and as a complication of chronic vagus nerve stimulation [202]. In children, cough associated with ACE inhibitors resolves within days (3–7 days) after withdrawing the medication [198,199] and may not recur when the medication is recommenced [199]. The package insert for omeprazole includes cough as an adverse event in 1.1% of adults and a single case report was recently published [203] but no reports on children were found.

##### Otogenic causes – Arnold's ear-cough reflex

In approximately 2.3–4.2% of people (bilateral in 0.3–2%), the auricular branch of the vagus nerve is present and the Arnold's ear-cough reflex can be elicited [204-206]. Case reports of chronic cough associated with ear canal stimulation from wax impaction and cholesteatoma have been reported [207,208]. In children, the significance of the ear reflex and cough was described as early as 1963 [209] although recently reported again [210].

## Management options of non-specific cough

Cough is subject to the period-effect (spontaneous resolution of cough) [211] and thus non-placebo controlled intervention studies have to be interpreted with caution [212]. If any medications are trialled, a 'time to response' should be considered and considerations given to patient profile and setting (eg community practice vs tertiary hospital practice). The same empirical therapy (for asthma, GOR, and PND) suggested in adults [33] is largely inappropriate in children.

### Physician and parental expectations

Providing parents with information on the expected time length of resolution of acute respiratory infections may reduce anxiety in parents and the need for medication use and additional consultation [120]. Appreciation of specific concerns and anxieties, and an understanding of why they present are thus important when consulting children with non-specific cough. Educational input is best done with consultation about the child's specific condition [213]. A RCT [214] examining the effect of a pamphlet and a videotape promoting the judicious use of antibiotics, found that their simple educational effort was successful in modifying parental attitudes about the judicious use of antibiotics.

### Over the counter cough medications and anti-histamines

In contrast to adults where OTC medications, in particular codeine and its derivatives have been shown to be useful, systemic reviews for children have concluded that cough OTCs have little, if any, benefit in the symptomatic control of cough in children [215,216]. Moreover OTCs have significant morbidity and mortality [217,218] and are common unintentional ingestions in children aged <5 years [219]. Use of diphenhydramine is also non beneficial for symptomatic treatment of cough related to pertussis [220]. The use of steam inhalation, vitamin C, zinc, and echinacea for upper RTI has been summarised [221] with little benefit, if any, for symptomatic relief of cough for adults and children.

The efficacy of anti-histamines in relieving cough in children is minimal, if at all [222]. Thus, unlike adults, the use of anti-histamine therapy for chronic cough in children is mostly unjustified. Whether this difference between children and adults is related to atopic states is unknown. A RCT on ketotifen did not show any clinical benefit in the treatment of 113 infants and children with chronic cough and/or wheeze [223]. A systematic review of anti-histamine and nasal decongestion combinations, and anti-histamines in OTC medications has shown that these pharmaceuticals were no more likely than placebo in reducing acute cough in children [215]. The use of these medications that contain H_1 _receptor antagonist has to be balanced with adverse events [217,224,225] which includes reported death from toxicity in young children [217,218]. The latest published RCT (n = 100) also showed that diphenhydramine (a first generation H_1_-antagonist) and dextromethorphan were no different to placebo in reducing nocturnal cough or sleep disturbance in both the children and parent(s) [225]. Like other RCTs there was a significant improvement in both placebo and active arms for the cough outcomes measured [225].

### Asthma therapy for cough

Old cohort studies describing that asthma therapy for that era (oral orciprenaline, salbutamol syrup [226,227] theophylline [97,227] and metaproterenol with theophylline) [88] was useful in abolishing cough included children with clinically recognisable asthma. For example, 8 of 11 children in Konig's study had cough with co-existant chest pain or dyspnea on exertion) [88] 10 of 32 children in another study had abnormal examination findings [89].

In ambulatory children with acute cough (1–10 days) with no history of asthma and a normal chest examination, oral albuterol was not effective in reducing cough frequency or duration [48]. In a meta-analysis, Smucy et al likewise concluded that "there is no evidence to support using beta2-agonists in children with acute cough and no evidence of airflow obstruction" [228]. There is only one study on use of inhaled salbutamol in chronic cough (median of 8-weeks) which also showed no benefit [47]. There is no evidence for the use of anti-cholinergics for in children with non-specific cough [229]. Use of bronchodilators must be weighed against adverse events (eg tremor, irritability [48] behaviour change, cost).

Only 2 RCTs on ICS for chronic non-specific cough in children have been published and both groups have cautioned against prolonged use of ICS [46,47]. There is no RCT on oral steroids for non-specific cough in children. In cough associated with pertussis, dexamethasone provides no significant benefit for the symptomatic relief of cough [220]. Even in children with wheeze, a RCT found that oral steroids may confer no benefit [230]. In contrast to high doses used in adults, low dose ICS has been shown to be effective in the management of the majority of childhood asthma [231-233] and there were reported significant adverse events on high doses [234,235]. Thus if a trial of asthma therapy is ever warranted, use of a moderate dose (400 mcg/day equivalent of budesonide) is suggested. This practice is however discouraged in most settings. As the earlier studies in adults and children that utilised medications for asthma for the era reported that cough related to asthma completely resolved by 2–7 days [88,89,97,236] it is recommended that reassessment is done in 2–3 weeks. Cough unresponsive to ICS should not be treated with increased doses of ICS. Cough that resolves with ICS use may be related to the period effect (spontaneous resolution) [211] or a transient effect responsive to ICS use (ICS may also impact on non-asthmatic airways with pulmonary toxicants [237]). Thus clinicians should be cognisant that the child that appears to respond to ICS does not necessarily have asthma and the child should be re-evaluated off asthma treatment.

Cromoglycate and nedocromil reduces cough associated with asthma [238,239] and in children born prematurely [240]. An open, single arm trial reported significant reduction in cough scores from 30 to 15/week after 2-weeks of treatment with nedocromil (4 mg qid) with no additional benefit in subsequent 4-weeks [241]. There are no published RCTs [242]. Leukotriene receptor antagonists have been examined in adults for cough [243] but there is no RCT data in children. Theophylline utilized in old studies [97,227] may have an effect on cough separate from its 'anti-asthmatic' properties but there are no RCTs in children [244] and theophylline has a narrow therapeutic range. Oral theophylline, but not placebo, induced complete remission in adults with ACE inhibitor related cough [245]. There is a need for RCTs examining the effectiveness of theophylline for non-specific cough in children.

### Anti-microbials

The American Academy of Family Physician's guidelines discourages use of antibiotics except when rhinosinusitis and cough are present and not improving after 10 days [246]. Meta-analysis on anti-microbials for acute bronchitis (recent onset of productive cough without chronic obstructive pulmonary disease, sinusitis or pneumonia) in older children (aged >8 years) and adults showed a small benefit of 0.58 days but with significantly more adverse events [221]. In subacute cough, two paediatric RCT have shown that anti-microbials (amoxycillin/clavulanic acid [247] and erythromycin [248]) were more likely to achieve 'clinical cure' and also prevented progression of illness defined by need for antibiotics [249]. The quality of cough in both studies was not clearly defined but the secretions in both studies cultured M catarrhalis [247,248].

### Cessation of ETS and other environmental toxicants

In the management of any child with cough irrespective of the aetiology, attention to exacerbation factors is encouraged. A single report was found on cessation of parental smoking as a successful form of therapy for the children's cough [250]. Behavioural counselling for smoking mothers has been shown to reduce young children's ETS exposure in both reported and objective measures of ETS [251].

## Conclusion

Cough is very common and in the majority is reflective of expected childhood respiratory infections. However cough may also be representative of a significant serious disorder and all children with chronic cough should have a thorough clinical review to identify specific respiratory pointers. Physiologically, there are similarities and significant differences between adults and children. Expectedly, the aetiologies and management of cough in a child differ to those in an adult. Cough in children should be treated based on aetiology and there is no evidence for using medications for symptomatic relief of cough or for an empirical approach based on the big three adult aetiologies. The use of medications are discouraged based on current evidence and if medications are used, it is imperative that the children are reviewed within the time frame of 'time to response' and medications ceased if there is no effect. Irrespective of diagnosis, environmental influences and parental expectations should be reviewed and managed accordingly as cough impacts on the quality of life of parents and children. Children with cough should be managed differently to adults as the aetiological factors and treatment in children differ to those in adults.

## Abbreviations

ACE Angiotensin converting enzyme

AHR Airway hyper-responsiveness

ARI Acute respiratory infection

CRS Cough receptor sensitivity

CXR Chest X-Ray

CT Computed Tomography

ETS Exposure to tobacco smoke

FTT Failure to thrive

GOR Gastroesophageal reflux

HRCT High resolution computed tomography of the chest

ICS Inhaled corticosteroids

OTC Over the counter

eNO exhaled nitric oxide

QOL Quality of Life

RCT Randomised controlled trial

PCR Polymerase chain reaction

## Competing interests

No actual or potential conflict of interest exists.

AB Chang is funded by the Australian National Health Medical Research Council and the Royal Children's Hospital Foundation
